# Leslie Topp, *Freedom and the Cage: Modern Architecture and Psychiatry in Central Europe, 1890–1914*

**DOI:** 10.1093/shm/hkz010

**Published:** 2019-02-18

**Authors:** Sarah Marks

**Affiliations:** Birkbeck, University of London

In 1909, the Franz Josef I Provincial Institution for the Mentally Ill opened its doors in the Czech Moravian town of Kroměříž. The asylum consisted of a series of pastel-shaded villas housing patient wards set amongst conifers, expansive therapeutic gardens for agricultural therapy and a domed chapel dedicated to Cyril and Methodius, the patron saints of Slavic peoples. Aesthetically, it was—and remains—every bit as beguiling as the town’s adjacent ornamental flower gardens and the nearby Archbishop’s Palace. But the building of the Kroměříž psychiatric hospital was not only a local, Moravian undertaking: designed by the Austrian architect Hubert Gessner and inspired by Vienna’s Steinhof asylum, but it was also part of a wider story about modernism, politics and psychiatry in the late Austro-Hungarian Empire. Leslie Topp’s visually appealing, large-format monograph uncovers the layered archival microhistories of seven Habsburg psychiatric institutions: located in Vienna and Mauer-Öhling in Austria, Prague and Kroměříž in Bohemia and Moravia, respectively, Krakow in Galicia and Gorizia and Trieste in present-day Italy. All designed and constructed between 1890 and 1914, the stories of their architectural conception and realisation reveal complex negotiations between local and regional officials, psychiatrists and the architects themselves, the latter of whom were some of the most prestigious in the region at that time. The book, therefore, sits at the intersection of two of the key fields grappling with questions of European modernity: the history of psychiatry and the mind and the history of architecture and design. As numerous other historians have noted, Central Europe itself was a crucible for innovation in both.

Drawing from imperial, regional and municipal archives, the book showcases maps, site plans, architectural models and plentiful photographs of both the interiors and the surrounds of the realised hospitals, along with their patient inhabitants and staff. Architectural styles were negotiated locally. For Vienna’s Steinhof, designed by the arch-Secessionist Otto Wagner, responsible for much of the city’s architecture from that period, the intellectual background was ‘Nietzsche, *Lebensreform* and the *Gesamtkunstwerk* – a context in which a community built from scratch was both a tool and a symbol of cultural regeneration’ (p.73). At Trieste, despite a competition having been held open to architects from across the Austro-Hungarian and German empires, the project was awarded to a local, Italian candidate, who drew upon classical Roman motifs, playing to the sensibilities and traditions of the region.

The real theoretical weight of the monograph comes through in Topp’s analysis of the spatial arrangement of the hospitals themselves and the political and clinical discussions that shaped them. Isolation in individual rooms, for example, for some was seen as a restriction of freedom and community; for others, it was a progressive move away from the chaotic corridor formations of older asylums. And for others, still it represented a means of enabling a degree of freedom for the patient without the need for mechanical restraint. Such debates from the period are carefully traced for each asylum under consideration. These are then placed in a wider framework drawing from Michel Foucault’s own concept of the patient’s ‘caged freedom’ within the institution (from which the book takes its title) and Patrick Joyce’s work on modernity and the built environment as fulfilling the dual, if somewhat contradictory, function of enabling freedom through providing a structured rule of order. Regulations and limited constraints within the asylum—as in the growing cityscapes of the fin-de-siècle—were introduced with the very aim of facilitating the possibility of a liberated life.

The author’s focus on landscape and the built environment eschews detailed narratives about treatment, except where these considerations informed or were influenced by spatial concerns. A more multidimensional exploration of life and practice within the walls of the asylums once they were built would have made an even richer contribution, but this is a separate project to the one this book aims to achieve. This absence does bring into sharp relief how little has been written, still, about psychiatric practice and patient experience in the empire and its successor states—the secondary literature, in most cases, simply does not yet exist, and the author has made the best use of what is available. And by looking at psychiatry from a different viewpoint this book raises wider questions about the relationship between environment, space and mental health, all of which are central concerns for current scholarship—notably featured in the Wellcome Collection’s recent exhibition, Living with Buildings and a broader ‘spatial turn’ in the history of science.

Topp’s reconstruction of the debates about the design and funding of these institutions, and their important status in their respective regions of the Habsburg Empire, invites us to reflect on how such concerns have changed or indeed diminished in the century since their establishment. The drive towards deinstitutionalisation in parts of Europe was itself associated with two of these very asylums, Trieste and Gorizia. Psychiatrist Franco Basaglia, who worked in both in the 1960s and 70s, led the movement for the abolition of mental hospitals in Italy—which was subsequently taken up by others across western Europe. In many other parts of the former Habsburg Empire—the Czech, Hungarian and Polish lands in particular, large psychiatric institutions are still a mainstay of mental healthcare, although efforts towards reform and deinstitutionalisation have been set in motion. In contrast with the turn of the previous century, the ideological project of the modernist asylum has all but disappeared in contemporary psychiatric discourse and perhaps along with it, sustained reflection on how buildings, institutions and physically constituted communities of patients might have psychotherapeutic value. But there are some remnants: the psychiatric hospital at Kroměříž, for example, still functions as an in-patient facility for Moravia, along with a dedicated villa that functions as a short-stay therapeutic community for patients suffering from neuroses, offering therapeutics ranging from psychodrama and art therapies to self hypnosis—all housed within the same architectural surrounds designed in the 1900s. All such modalities could be offered within the community, but for the practitioners there, the residential aspect of this facility remains key to the treatment programme. The histories presented in *Freedom and the Cage*, therefore, are all the more timely as Central European societies consider the reform of mental health care and the role that institutional hospitals may continue to have into the 21st century.

